# Periocular skin warming elevates the distal skin temperature without affecting the proximal or core body temperature

**DOI:** 10.1038/s41598-019-42116-x

**Published:** 2019-04-05

**Authors:** Tomohisa Ichiba, Masahiro Suzuki, Sayaka Aritake-Okada, Makoto Uchiyama

**Affiliations:** 1 Personal Health Care Laboratory, Kao Corporation, 2-1-3, Bunka, Sumida-ku, Tokyo 131-8501 Japan; 2 Department of Psychiatry, Nihon University School of Medicine, 30-1, Oyaguchi Kamicho, Itabashi-ku, Tokyo 173-8610 Japan; 3 Faculty of Health and Social Services, Saitama Prefectural University, 820, Sannomiya, Koshigaya, Saitama 343-8540 Japan

## Abstract

Periocular skin warming reportedly improves the objective and subjective sleep quality in adults with mild difficulty in falling asleep. To clarify the effects of periocular warming, we examined the distal skin temperatures (hands and feet), proximal skin temperature (infraclavicular region) and core body temperature as well as the distal-proximal skin temperature gradient (DPG). Nineteen healthy males underwent two experimental sessions, wherein they used a warming or sham eye mask under a semi-constant routine protocol in a crossover manner. Participants were instructed to maintain wakefulness with their eyes closed for 60 minutes after wearing the eye mask. The warming eye mask increased the periocular skin temperature to 38–40 °C for the first 20 minutes, whereas the temperature remained unchanged with the sham mask. Compared to that of the sham eye mask, the warming eye mask significantly increased the temperatures of the hands and feet and the DPG, whereas the proximal skin and core body temperatures were unaffected. Subjective sleepiness and pleasantness were significantly increased by the warming eye mask. These results represent physiological heat loss associated with sleep initiation without affecting the proximal skin or core body temperatures, suggesting that thermal stimulation in certain areas can provoke similar changes in remote areas of the body.

## Introduction

Sleep is reported to be closely related to the circadian rhythm of the core body temperature (CBT)^1,2^. The circadian rhythm of the CBT is modulated by heat production and loss via the distal skin temperature^3,4^. Therefore, the circadian rhythm of the distal skin temperature in the feet and hands exhibits an inverse pattern to that of the CBT^3,4^. Heat loss is indirectly measured by assessing the temperature gradient from the proximal (i.e., infraclavicular, thigh, and stomach) skin to the distal (i.e., foot and hand) skin, which is known as the distal-proximal skin temperature gradient (DPG). The DPG is reportedly a good predictor of sleepiness and is correlated with sleep onset latency^5,6^.

Previous studies have indicated that manipulation of the skin or body temperature can enhance or disturb sleep in humans^7–9^. One study demonstrated that passive body heating increased heat loss and improved subjective sleep initiation^7^. Other studies also documented that warming of the feet or hands improved sleep quality^8,9^. Conversely, clinical studies have indicated that cold feet or hands due to vasoconstriction syndrome are associated with poor sleep quality^10–12^. Pharmacological studies have revealed that administration of melatonergic agents^13,14^ and benzodiazepine hypnotics^15^ also enhances heat loss via distal skin regions and induces sleep. Thus, direct warming manipulation of the skin temperature and hypnotic drug administration are likely to modulate sleep by controlling heat loss processes for skin regions.

We developed a disposable heat- and steam-generating sheet (HSG) to safely and easily warm local skin regions and induce mental and physical relaxation (e.g., the periocular^16–18^, neck^17,19^, and abdominal skin regions^20^). Recently, warming of the periocular skin at bedtime has been reported to improve the subjective sleep quality^16,17^ and increase the delta power during sleep^17^ in adults with mild difficulty falling asleep. Previous observations that warming the abdominal skin region increases the skin blood flow and temperature in the feet and fingers^20^ may indicate that periocular skin warming influences sleep through a similar heat loss process via distal skin regions, as reported in previous clinical and intervention studies.

In the present study, we warmed the periocular skin region using a previously developed eye mask with a HSG^16–20^, and evaluated the skin and core body temperatures after application of the eye mask. To minimise the confounding effects of sleep, posture, food intake, and ambient temperature on body temperature, we utilised a semi-constant routine (CR) protocol developed by Wright Jr. *et al*.^14^. We aimed to find differential effects of periocular skin warming on the distal and proximal skin and core body temperatures.

## Results

Participants were instructed to maintain wakefulness with their eyes closed for 60 minutes after wearing the warming or sham eye mask in a crossover manner. Polysomnographic (PSG) recordings, including electroencephalography (EEG), electrooculography (EOG), and electromyography (EMG), were scored at every 30-s period during the observation period, and the participants were determined to be in the stage wake according to standard criteria, which ensured that the maintenance of wakefulness was successful in the present experimental procedure.

### Subjective ratings

**Figure 1:**
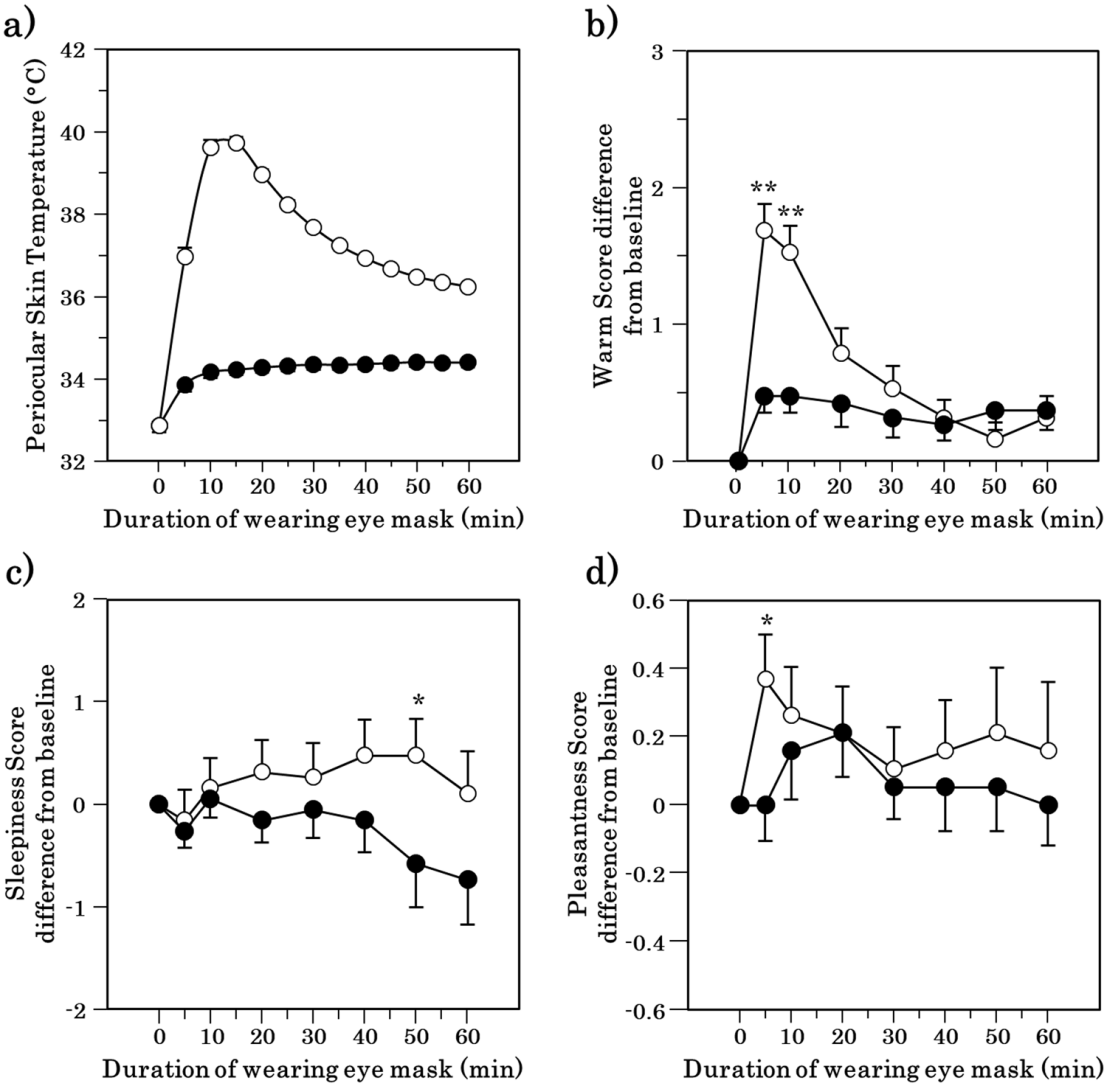
Time course of periocular skin temperature and Changes in the subjective ratings after the periocular warming (open circle) or the sham condition (closed circle). (**a**) Periocular skin temperature. (**b**) The subjective warmth around the periocular region. (**c**) The subjective sleepiness. (**d**) The subjective pleasantness. All values are means ± s.e.m. Comparisons performed relative to sham condition using Wilcoxon signed rank test. *p < 0.05, **p < 0.01.

The warm sensation around the periocular region, sleepiness, and pleasant feeling ratings obtained during the semi-constant routine are shown in Fig. 1. The ratings for the warm sensation around the periocular region were significantly higher at 5 and 10 min after the initiation of wearing the warming eye mask than for wearing of the sham mask (p = 0.001 and p = 0.002, respectively; Fig. 1a). Significant differences were found between periocular warming and the sham condition with respect to sleepiness at 50 min (p = 0.034; Fig. 1b) and a pleasant feeling 5 min after application (p = 0.021; Fig. 1c).

### Body temperature

**Figure 2:**
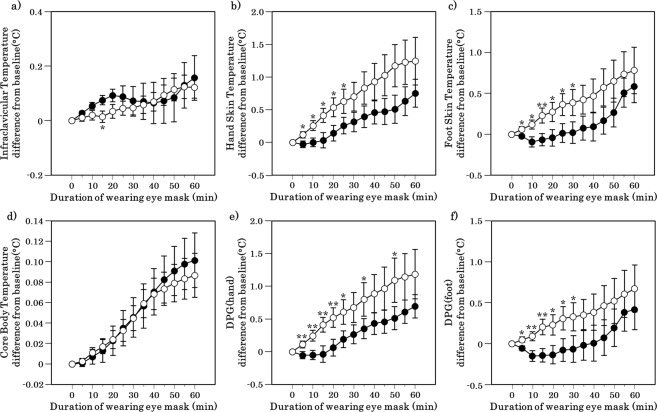
Time course of core body temperature, skin temperature and DPG after the periocular warming condition (open circle) and the sham condition (closed circle). (**a**) Infraclavicular temperature. (**b**) Hand skin temperature. (**c**) Foot skin temperature. (**d**) Rectal (core) temperature. (**e**) DPG (hand). (**f**) DPG (foot). All values are means ± s.e.m. Comparisons performed relative to sham condition using Wilcoxon signed rank test. *p < 0.05, **p < 0.01.

The T_foot_ and T_hand_ were significantly elevated during the first half of the application under the periocular warming condition compared with that under the sham condition (the exact p values are presented in Supplementary Table S1; Fig. 2b,c). A significant difference was found at 15 min in the infraclavicular temperature between the periocular warming and sham conditions (p = 0.044; Fig. 2a).

The DPG_hand_ was significantly higher under the periocular warming condition than under the sham condition at 5, 10, 15, 20, 25, and 35 min after application (the exact p values are presented in Supplementary Table S1; Fig. 2e). The DPG_foot_ was significantly elevated under the periocular warming condition compared with that under the sham condition at 5, 10, 15, 20, 25, and 30 min after application (the exact p values are presented in Supplementary Table S1; Fig. 2f). The T_core_ did not differ between the two conditions over the course of the observation period (Fig. 2d).

### EEG and Heart rate variability

**Figure 3:**
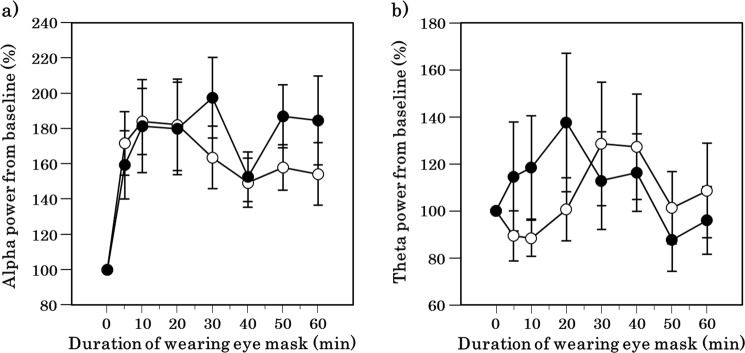
Time course of alpha and theta power spectrum after the periocular warming condition (open circle) and the sham condition (closed circle). (**a**) Change in alpha power for baseline. (**b**) Change in theta power for baseline (an index of cardiac sympathetic activity). All values are means ± s.e.m. Comparisons performed relative to sham condition using Wilcoxon signed rank test.

Alpha and theta power did not differ between the periocular warming and sham conditions over the course of the observation period (Fig. 3).

**Figure 4:**
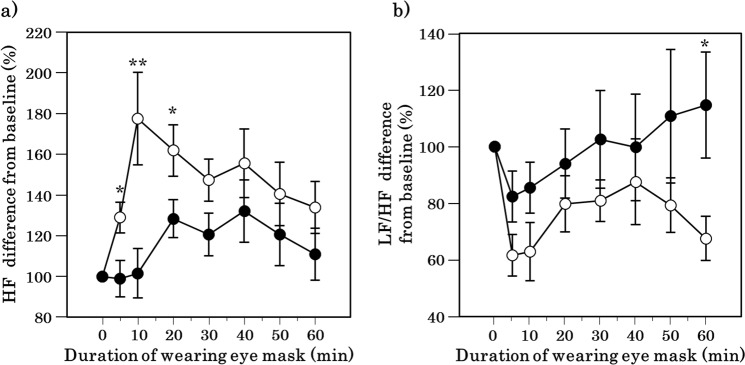
Time course of heart rate variability after the periocular warming condition (open circle) and the sham condition (closed circle). (**a**) Change in the rate of high frequency (HF) for baseline (an index of cardiac parasympathetic activity). (**b**) Change in the rate of the ratio of low frequency to high frequency (LF/HF) for baseline (an index of cardiac sympathetic activity). All values are means ± s.e.m. Comparisons performed relative to sham condition using Wilcoxon signed rank test. *p < 0.05, **p < 0.01.

Heart rate variability (HRV) was measured to observe changes in the cardiac autonomic function. Changes in the high frequency of the HRV during application of the eye mask were significantly higher at 5, 10, and 20 min in the HSG than in the sham condition (p = 0.028, p = 0.005, and p = 0.022, respectively; Fig. 4a). Furthermore, changes in the ratio of low frequency to high frequency were significantly lower at 60 min in the periocular warming condition than in the sham condition (p = 0.013; Fig. 4b).

## Discussion

In the present study, which used a semi-CR protocol to minimise the possible effects of sleep, posture, movement, food intake, and ambient temperature on skin and body temperature measurements, we found that periocular skin warming increased the temperatures in the distal hand and foot skin but did not affect the truncal skin or core body temperature. During the warming procedure, the participants reported a warm sensation in the periocular skin region, subjective sleepiness and pleasant feelings and showed HR variability changes.

First, we showed in the present study that periocular skin warming enhanced sleepiness and pleasant feelings. This result may be comparable with previous findings that warming the dorsum of the hand increases pleasant feelings in healthy humans compared to the effects of cooling^21^. In the present study, pleasant feelings were significantly enhanced only for the first 10 min of the periocular skin warming period in parallel with the warm sensation in the periocular region, suggesting that the warm sensation in the periocular skin region was associated with the pleasant feelings. Although no difference was found in the warm sensation in the second half of the session, subjective sleepiness emerged only during the second half. This result indicated that the pleasant feelings might have been an initial response and that sleepiness was a subsequent subjective response to periocular warming.

The enhancement of subjective sleepiness in the later part of the periocular warming period in the present study, which occurred after the warm sensation was reduced, suggested that the effects on sleep might be mediated through physiological changes other than immediate elevation of the local skin temperature. This notion also provides a potential interpretation of the effects of bedtime periocular warming on subsequent nocturnal sleep^16,17^. We previously reported that warming the periocular skin region before bedtime with the eye- mask improved the subjective quality of subsequent nocturnal sleep^16^ and increased the sleep delta activity during the first 90-min period^17^, in subjects with mild sleep problems. Further, in this study, the increase of distal skin temperature and the enhancement of subjective sleepiness by periocular warming in the daytime were observed. Taken together, effects of periocular warming observed in our previous studies^16,17^ might have been associated with similar physiological changes found in the present study conducted during the daytime.

One of the most remarkable findings obtained in the present study was that periocular warming was associated with a significant skin temperature elevation in the feet and hands, which are regions remote from the periocular region. The present study was carefully designed in a random crossover manner methodologically by applying a semi-CR protocol to minimise confounding factors such as sleep, posture, movement, food intake and ambient temperature, which were previously reported to affect skin temperature^14^. Regarding the mechanisms of remote skin temperature elevation by outside intervention in the present study, direct heat conduction through the skin did not appear to be a likely mechanism, because neither the truncal skin temperature nor the core body temperature was influenced by the periocular warming. A more likely explanation was that the periocular skin warming influenced the skin temperature in remote regions through central nervous system controls. The periocular warming may have induced changes in the autonomic centre in the brain or spinal cord, which elevated the skin temperature in the feet and hands. The immediate change in the skin temperatures in the feet and hands in parallel with changes in the subjective warming sensation may support neuronal control rather than direct heat conduction over the skin.

Local skin warming in the foot or hand was not reported to be associated with an increase in the core body or skin temperature of other parts of the targeted region^22,23^. Furthermore, whole passive body warming was documented to increase both the core body and skin temperatures^7,24^. Only one study has demonstrated a skin temperature increase in the feet and hands after local skin warming in the abdominal or lumbar region^20^. However, these authors did not refer to the phenomena or confirm its possible mechanism. The present study was the first to show that periocular skin warming selectively elevated skin temperatures in the feet and hands.

Increased local skin temperature in the foot or hand has been reported to be associated with distal vasodilation controlled by the autonomic nervous system^25^, is one of the most robust signs of heat loss, and has been interpreted as a reliable preceding marker of a subsequent CBT decrease and sleep initiation^26^. In a previous study^27^, the reduction in CBT occurred a few hours after the distal skin temperature started to increase in physiological nocturnal sleep settings; however, studies focusing on naps in the daytime did not reveal a significant CBT reduction in association with sleepiness or distal skin temperature elevation^27^. Our study finding that changes in CBT were not observed during a 60-min experimental period was comparable to that of previous studies conducted in similar settings^27^. The difference in changes in the CBT before and after sleep initiation may be accounted for by the shorter observation period in the nap studies than in nocturnal sleep studies. In previous studies^28,29^, increased distal skin temperature and reduction in CBT were caused almost simultaneously by melatonin administration, suggesting that heat loss from skin surface may have immediately led to the decrease of CBT. In the present study, however, the increase in distal skin temperature and DPG that occurred due to periocular warming in the early phase of the observation period was not associated with changes in CBT, though there was a slight non-significant decrease of CBT (approximately 0.01 °C shown in Fig. 3d). Therefore, the present findings may indicate that periocular warming has thermoregulatory effects that are different from those caused by physiological or pharmacological intervention used in the previous studies^28,29^.

In the present study, periocular skin warming was associated with an increase in the high frequency power and decrease in the ratio of low frequency to high frequency in the HRV analyses. Studies have indicated that the high frequency power value may represent the intensity of parasympathetic activity and that the ratio of low frequency to high frequency may represent the sympathetic activity intensity. Given the functional significance of heart rate variability, periocular skin warming may have increased parasympathetic but decreased sympathetic activities. One study has documented that periocular skin warming is associated with a simultaneous decrease in hemodynamic activity in the dorsolateral prefrontal cortex, potentially indicating relaxation^18^. Previous studies have noted a relationship between intraocular pressure and local temperature^30^. Increases in ocular pressure have been shown to reduce the heart rate or oculocardiac reflex^31^. Therefore, we could assume that changes in intraocular pressure might have mediated the present findings.

This study had several limitations. First, subjective evaluations on sleepiness and pleasant feelings changed during the periocular warming, which were not associated with EEG alterations in alpha and theta power. The discrepancy between subjective and objective measures should be investigated in a future study. Second, although the participants could not distinguish the appearance between the sham and warming eye masks, they were able to distinguish the warm condition from the sham condition. Third, the warming eye mask in this study kept the periocular skin at 38–40 °C for approximately 20 min. The duration of warming in the present study was different from that of the previously reported eye mask^17^ which warmed the periocular skin to 38–40 °C within approximately 10 min. Therefore, clarifying the relationship between the warming duration and the changes in skin temperature may be necessary. Fourth, although our previous reports suggested some improvement of sleep quality by periocular warming^16,17^, the present results alone may not sufficiently guarantee that periocular warming improves nocturnal sleep. This is because the present study was conducted in the daytime. Further studies under nocturnal sleep conditions are required to confirm the direct relationship between periocular warming and nocturnal sleep.

In conclusion, we documented for the first time that periocular skin warming increased the distal skin temperature in the feet and hands, which was one of the most robust factors predicting subsequent sleep.

## Methods

### Participants

Twenty healthy men aged 29–57 years (mean ± SD; 45.8 ± 7.7) who were free of any medical conditions were recruited through a clinical research organisation. None of the participants had a current or prior history of physical, mental, or sleep disorders or hypnotic medication use. Those who engaged in shift work or whose habitual sleep duration was <5 h or >9 h were not included. Ethical approval was obtained from the Ethics Committee of Kao Corporation, and written informed consent was obtained from all of the participants after they received a detailed explanation of the experiment according to the Declaration of Helsinki. One participant was excluded because he went to the restroom during the experimental sessions and the measurements were not completed. Nineteen participants completed the entire study.

### Experimental procedures

The participants underwent two experimental sessions separated by one week in a randomised, crossover, placebo-controlled design. One session was set as the “warm session” and the other as the “sham session”. During the warm session, the participants used a warming eye mask with HSG; in the sham session an eye mask with an inactivated HSG was used. Each experimental session was performed on the same day of the week in the laboratory. The participants were instructed to maintain their habitual sleep-wake schedule for 1 week before the session, which was verified by a sleep diary and an activity monitor used for activity-based sleep recordings (MTN210; Kissei Comtec Co., Ltd, Nagano, Japan). They were asked to refrain from alcohol and caffeinated beverages for 24 h before the experimental session.

**Figure 5:**
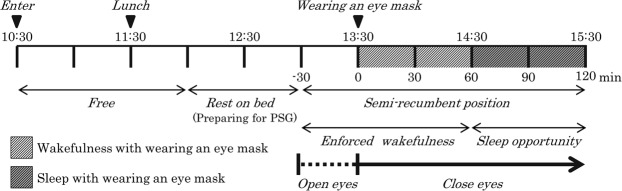
Experimental session.

A schematic explanation of the experimental session is shown in Fig. 5. The session consisted of an adaptation period and a semi-CR protocol in which the participants were kept in a semi-recumbent position under dim light (~100 lux) and constant ambient temperature (21–22 °C). The participants were admitted to the laboratory at 10:30, wore light clothing, and stayed there until 11:30 while quietly reading books or listening to music. The use of cellular phones or digital monitors was prohibited throughout the experimental session. An iso-caloric (500~600 kcal) lunch box was provided at 11:30. Then, to prepare for PSG and the temperature recordings, the participants were instructed to maintain a semi-recumbent posture on a bed with their eyes open and their head raised at ~35 degrees at 13:00. Throughout the periods of enforced wakefulness and sleep opportunity, they were covered with a sheet. The eye mask was placed on the periocular region by research assistants at 13:30. The participants were instructed to maintain wakefulness with their eyes closed until 15:30. The research assistants monitored the participants’ states visually and via EEG recordings to ensure the enforced wakefulness. The EEG, EOG and EMG recordings obtained during the enforced wakefulness were later scored at 30-s periods according to standard criteria^32^. Thereafter, the participants were given a 1-h sleep opportunity in the same posture.

### Eye masks

**Figure 6:**
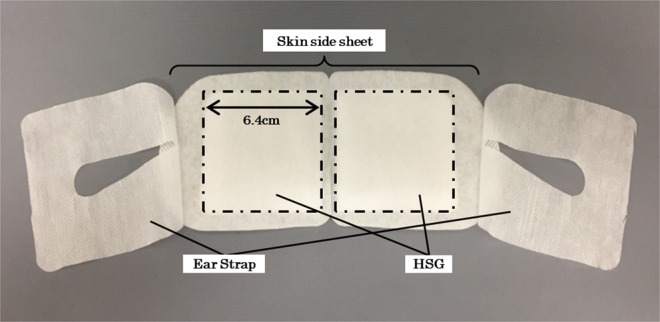
Photographic illustration of the warming eye mask.

The warming eye mask used in the present study was made of nonwoven fabric and had a disposable HSG that provided moist heat through the chemical reaction of iron, water, and oxygen when its package was opened. The warming eye mask increased the periocular skin temperature gradually to 38 °C within approximately 5 min and maintained the temperature at 38–40 °C for approximately 20 min. The sham eye mask was an inactivated HSG made of the same nonwoven fabric and was indistinguishable from the warming eye mask but did not provide moist heat. The periocular skin temperature remained unchanged with the sham eye mask. The eye masks were placed to cover both eyes and the periocular region and ensure that all outside vision was cut off. The eye masks were the prototype made by Kao Corporation for the present study (Fig. 6).

### Polysomnographic recordings

PSG recordings were performed with the Biosignal Amplifier system (Polymate AP216, Miyuki Giken Corp., Tokyo, Japan) using four EEG channels (Fz, Cz, Pz, and O1, referenced against linked mastoid A1 and A2), two EOG channels (right and left outer canthus), a submental EMG, and electrocardiogram (ECG) channels. The EEG, EOG, and ECG signals used a high-frequency filter of 60 Hz, a low-frequency filter of 0.15 Hz, and a 60-Hz notch filter. The high-frequency and low-frequency filters for EMG were set at 10 and 100 Hz, respectively. All data were sampled at 500 Hz.

### Analysis of EEG and heart rate variability

To evaluate the effect of the periocular warming on changes in EEG, the EEG signal (Cz-A1) was band-pass filtered between 0.5 and 40 Hz. Subsequently, alpha (8.0–12.0 Hz) and theta (4.0–7.0 Hz) power spectrum for consecutive 5-s periods were computed using a Fast Fourier Transform (FFT) with a Hamming window. Each power spectrum for 5-s periods was averaged over a 60-s period before the subjective ratings, and the baseline alpha and theta power spectrum were defined as the values collected during the 60-s period before applying the eye mask. Changes in alpha and theta power spectrum after wearing the eye mask were assessed by calculating the ratio of the value of each point to that at the baseline^33^.

The HRV analysis was performed according to the procedures described in a previous study^34^. The R-R interval (RRI) was detected from the ECG recordings, artifacts were removed, and the RRI times were linearly interpolated with the surrounding values. The spectral analysis of the RRI for the 3-min period was performed using FFT to obtain the powers in the low frequency (0.04–0.15 Hz) and high frequency (0.15–0.40 Hz) of the HRV. The ratio of low frequency to high frequency was calculated using the power in each band. The high frequency values and the ratio of low frequency to high frequency were assessed during the 3-min rest period before the subjective ratings, and the baseline high frequency and the ratio of low frequency to high frequency were defined as the values collected during the 3-min rest period before applying the eye mask. Changes in the high frequency and the ratio of low frequency to high frequency after wearing the eye mask were assessed by calculating the ratio of the value of each point to that at the baseline. Data from one participant were not used for the HRV analysis because of technical issues. These analyses were performed with the software package MATLAB (The Math Works Inc., USA) and its signal processing toolbox.

### Temperature recordings

Core body and skin temperatures were recorded using a portable device (LT-200, Gram Corp, Saitama, Japan) with a sampling rate of 1 Hz. The core body temperature (T_core_) was obtained using a thermistor that was self-inserted approximately 10 cm into the rectum. Thermistors to measure the skin temperature were fixed on the skin with thin, air-permeable, adhesive surgical tape (Fixomull stretch; BSN medical, Hamburg, Germany) in the following 7 body areas: the left and right sides of the foot (the middle of the dorsum side), hand (the middle of the dorsum side), infraclavicular regions and left eyelids. All temperature data were smoothed by a 5-min moving average and resampled in 5-min bins. The mean value of the left and right infraclavicular regions was defined as the proximal skin temperature (T_prox_). The foot skin temperature (T_foot_) and hand skin temperature (T_hand_) were averaged for both the foot and hand, respectively. To calculate the DPG, which was the difference between the T_foot_ and T_prox_, T_hand_ and T_prox_ were defined as DPG_foot_ and DPG_hand_, respectively.

### Subjective ratings

During the semi-CR, the subjects were instructed to report verbally their responses to the Karolinska sleepiness scale (KSS) and the subjective ratings regarding the pleasantness and intensity of the warmth around the periocular region when asked. The KSS consisted of a 9-point scale from 1 (very alert) to 9 (very sleepy). The pleasantness rating was a 5-point scale as follows; 1 = very unpleasant, 2 = unpleasant, 3 = neutral, 4 = pleasant, and 5 = very pleasant. The rating of the warmth intensity consisted of a 5-point scale as follows; 1 = neutral, 2 = slightly warm, 3 = warm, 4 = very warm, and 5 = hot. Subjective ratings were verbally performed three times during the 30 min before application of the eye mask. The participants were pre-trained how to respond verbally twice among the 3 rounds before application of the eye mask. Thereafter, they reported subjective evaluations at 5, 10, 20, 30, 40, 50, and 60 min after eye mask placement.

### Sample size

The sample size was calculated a priori using the G*Power version 3.1.9.2. We used a two-tailed Wilcoxon signed rank test with alpha level of 0.05, power of 0.90, and an effect size of 0.8. A sample size of 20 participants was planned. Since the present study was the first to investigate skin temperature after skin warming, the hypothesized effect size was postulated from a similar pharmacological intervention study^14^, in which a sample size of 14 was used. We used a total sample size of 20 because the effect of the physiological intervention used in this study was presumed to be more moderate than that of the pharmacological intervention.

### Statistical analyses

All values were expressed as the mean ± standard error of the mean (s.e.m.). All statistical analyses for comparisons between periocular warming and the sham conditions were performed with the Wilcoxon signed-rank test. Probability values of less than 0.05 were accepted as statistically significant. All statistical analyses were performed using IBM SPSS Statistics 20 (IBM, Chicago, IL, USA).

## Supplementary information

Supplementary Table S1

## Data Availability

The datasets analyzed during the current study are available from the corresponding author on reasonable request.
