# Supplementary material for: Periocular skin warming elevates the distal skin temperature without affecting the proximal or core body temperature
Source: Sci Rep. 2019 Apr 5;9:5743. doi: 10.1038/s41598-019-42116-x (PMC6450979; doi:10.1038/s41598-019-42116-x)
Supplement: Supplementary file 1 — Supplementary Table S1 [file 41598_2019_42116_MOESM1_ESM.docx]

**Supplementary Information**

Periocular skin warming elevates the distal skin temperature without affecting the proximal or core body temperature

Tomohisa Ichiba^1,2,†,*^, Masahiro Suzuki^2,†^, Sayaka Aritake-Okada^3^, Makoto Uchiyama^2,*^

^1^ Personal Health Care Laboratory, Kao Corporation, 2-1-3, Bunka, Sumida-ku, Tokyo 131-8501, Japan.

^2^ Department of Psychiatry, Nihon University School of Medicine, Oyaguchi Kamicho, Itabashi-ku, Tokyo 173-8610, Japan.

^3^ Faculty of Health and Social Services, Saitama Prefectural University, 820, Sannomiya, Koshigaya, Saitama, 343-8540, Japan

**^†^** These authors contributed equally to this work.

^*^Corresponding author: [ichiba.tomohisa@kao.com](mailto:ichiba.tomohisa@kao.com) and [uchiyama.makoto@nihon-u.ac.jp](mailto:uchiyama.makoto@nihon-u.ac.jp)

**Supplementary Table S1. *P* values for body temperatures**

|  | T_prox_ | T_hand_ | T_foot_ | DPG_hand_ | DPG_foot_ |
| --- | --- | --- | --- | --- | --- |
| 5 min | 0.376 | 0.013 | 0.024 | 0.009 | 0.024 |
| 10 min | 0.159 | 0.020 | 0.020 | 0.004 | 0.005 |
| 15 min | 0.044 | 0.013 | 0.007 | 0.002 | 0.003 |
| 20 min | 0.084 | 0.022 | 0.014 | 0.005 | 0.018 |
| 25 min | 0.198 | 0.044 | 0.024 | 0.010 | 0.022 |
| 30 min | 0.355 | 0.117 | 0.027 | 0.059 | 0.036 |
| 35 min | 0.355 | 0.077 | 0.171 | 0.033 | 0.091 |
| 40 min | 0.520 | 0.099 | 0.136 | 0.064 | 0.117 |
| 45 min | 0.573 | 0.099 | 0.107 | 0.064 | 0.227 |
| 50 min | 0.520 | 0.070 | 0.227 | 0.044 | 0.260 |
| 55 min | 0.314 | 0.091 | 0.376 | 0.077 | 0.398 |
| 60 min | 0.198 | 0.212 | 0494 | 0.147 | 0.494 |
